# Enhanced cytomegalovirus infection in human trabecular meshwork cells and its implication in glaucoma pathogenesis

**DOI:** 10.1038/srep43349

**Published:** 2017-02-27

**Authors:** Jin A Choi, Ju-Eun Kim, Seung-Jun Noh, Eun Kyoung Kim, Chan Kee Park, Soon-Young Paik

**Affiliations:** 1 Department of Ophthalmology, College of Medicine, St. Vincent’s Hospital, The Catholic University of Korea, Suwon, Republic of Korea; 2 Department of Microbiology, College of Medicine, The Catholic University of Korea, Seoul, Republic of Korea; 3 Research Institute of St. Vincent Hospital, College of Medicine, Catholic University of Korea, Suwon, Republic of Korea; 4 Department of Ophthalmology, College of Medicine, Seoul St. Mary’s Hospital, The Catholic University of Korea, Seoul, Republic of Korea

## Abstract

Cytomegalovirus (CMV) is one of the infectious causes of hypertensive anterior uveitis, which is characterized by recurrent episodes of elevated intraocular pressure (IOP) and mild anterior uveitis. Despite the potentially vision-threatening complications of this disease, the underlying mechanisms remain largely undefined. We aimed to investigate whether human trabecular meshwork (TM) cells, the key cell type that regulates IOP, could support CMV replication, as well as demonstrate the relevant pathological changes in TM. When human TM cells were infected with CMV AD169, immediate early antigens were detected 1 day post-infection (dpi); cytopathic changes including rounding, a ballooned appearance with disorganization, and a decreased number of stress fibers were noted in TM cells. The marked increase in viral DNA accumulation was observed most notably at 5 and 7 dpi, suggesting that the active viral infection in human TM cells could be the key mechanism underlying the elevation of IOP in anterior viral uveitis. Notably, CMV infection enhanced the production of transforming growth factor (TGF)-β1, an upstream molecule that increases the resistance of the outflow pathway in human TM cells. The increase of TGF-β1 was countervailed by additional treatment with corticosteroids. Our results provide a pathogenic mechanism for IOP elevation in viral anterior uveitis.

Anterior uveitis is the most frequent type of uveitis worldwide^1^. Infectious causes of anterior uveitis are increasingly being recognized, and the herpesviridae including herpes simplex virus (HSV), varicella zoster virus (VZV), and most recently, cytomegalovirus (CMV), have been demonstrated as causes of acute, recurrent, and chronic hypertensive anterior uveitis or corneal endotheliitis in immunocompetent patients^2^,^3^. The availability of PCR testing of aqueous humor has allowed viruses to be detected in conditions that were previously labelled idiopathic. The detection of viral DNA is used in clinical practice to diagnose herpes virus as the cause of some cases of anterior uveitis.

Anterior uveitis caused by herpes viruses is typically characterized by recurrent episodes of elevated intraocular pressure (IOP) and mild anterior chamber reactions with a few keratic precipitates^4^,^5^. The conventional treatment strategy for viral anterior uveitis involves the application of corticosteroids and anti-glaucoma agents, which can control inflammation and the elevation of IOP in most cases. However, with current conventional treatment, recurrence of the disease cannot be prevented, and repeated recurrence eventually leads to glaucoma in 8–24% of cases^5^,^6^. Moreover, a long duration of corticosteroid use is associated with steroid-induced glaucoma^7^.

Although a number of studies have reported the identification of herpes virus in aqueous samples from patients with hypertensive anterior uveitis^2^,^3^,^5^,^7^, the mechanism underlying the elevation of IOP induced by herpes virus has yet to be elucidated. Transforming growth factor-β (TGF-β) is found in increasing amounts in the aqueous humor of patients with primary open-angle glaucoma and is considered to be a cardinal cytokine that increases resistance in the trabecular meshwork (TM) outflow pathways^8^. *In vitro* infection of several cell cultures with HSV-1^9^ or CMV induce the secretion of TGF-β1^10^,^11^. CMV infection in renal allografts also accompanies higher levels of TGF-β1 compared with uninfected allografts^12^, and CMV infection of a renal graft is considered to accelerate the rejection of the graft via viral induction of TGF-β1 with resultant fibrosis^13^,^14^. In addition, significantly elevated TGF-β was noted in the aqueous humor of patients with hypertensive anterior uveitis compared with controls^15^. Among the herpes viruses, CMV, in particular, has been associated with severe corneal endothelial cell loss and higher requirements for glaucoma surgery^7^, both of which are vision-threatening complications. The condition may respond to ganciclovir but frequently relapses.

Regarding the elevation of IOP, human TM cells are considered to be a focus of inflammation in hypertensive anterior uveitis^16^. However, human TM cells supporting the fully permissive replication of human CMV has not been demonstrated. A model of CMV infection in human TM cells, the key cells involved in the regulation of IOP^17^, should provide insight into the mechanism of herpes virus-induced anterior uveitis. Therefore, in the present study, we determined whether human TM cells could support CMV replication and subsequently investigated whether CMV infection in human TM cells altered the expression of TGF-β1 and genes related to the extracellular matrix (ECM). Also, the effect of steroid and anti-viral agent, ganciclovir on CMV infection in TM cells was determined.

## Results

### Productive infection of CMV in human TM and HFF cells

**Figure 1:**
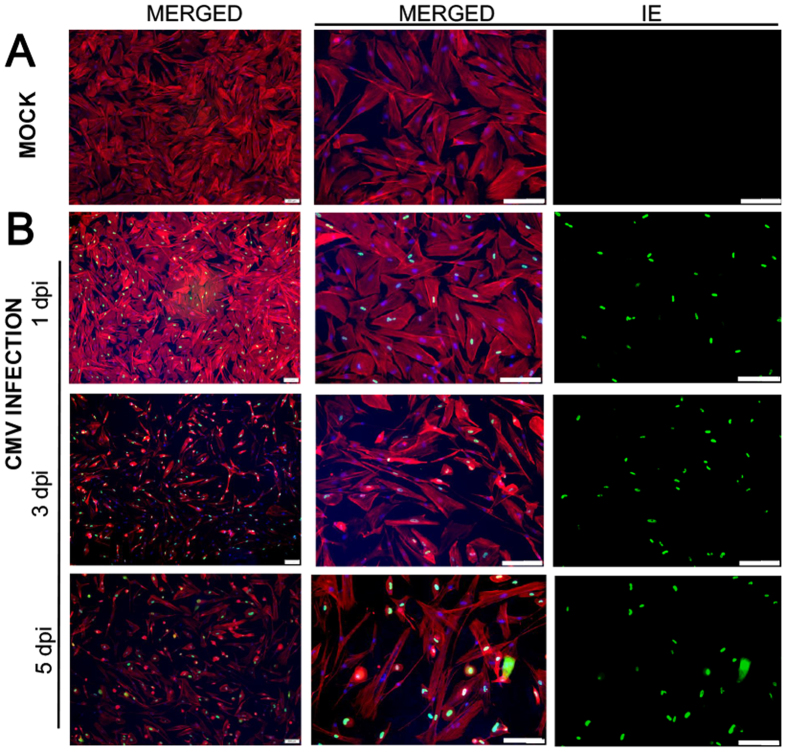
Expression of immediate-early (IE) antigen in the human trabecular meshwork (TM) cells after human cytomegalovirus (CMV) infection at multiplicity of infection of 1. Normal uninfected TM cells (**A**) and TM cells at 1, 3, 5 days post-infection (**B**). Nuclei were stained with DAPI (blue signals), IE antigen immunolabelled with a murine antibody were stained with an anti-mouse IgG secondary antibody conjugated with VectaFluor 488 (green signals), and stress fibers with a Rhodamine Phalloidin (red signals). Bar = 200 μm.

**Figure 2:**
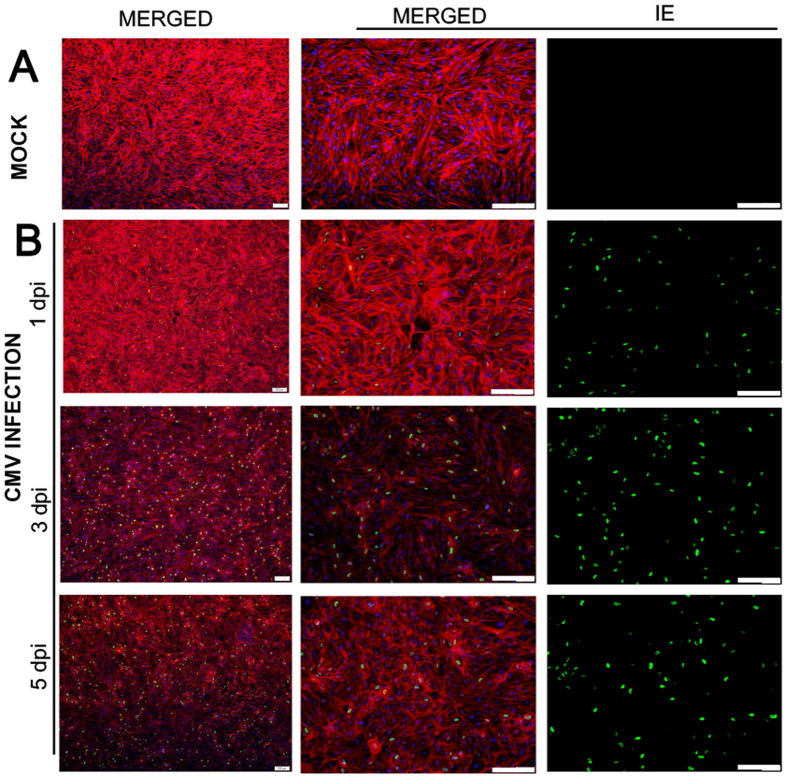
Expression of immediate-early (IE) antigen in the human foreskin fibroblast (HFF) cells after human cytomegalovirus (CMV) infection at multiplicity of infection of 1. Normal uninfected HFF cell (**A**) and HFF cell at 1, 3, 5 days post-infection (**B**). Nuclei were stained with DAPI (blue signals), IE antigen immunolabelled with a murine antibody were stained with an anti-mouse IgG secondary antibody conjugated with VectaFluor 488 (green signals), and stress fibers with a Rhodamine Phalloidin (red signals). Bar = 200 μm.

**Figure 3:**
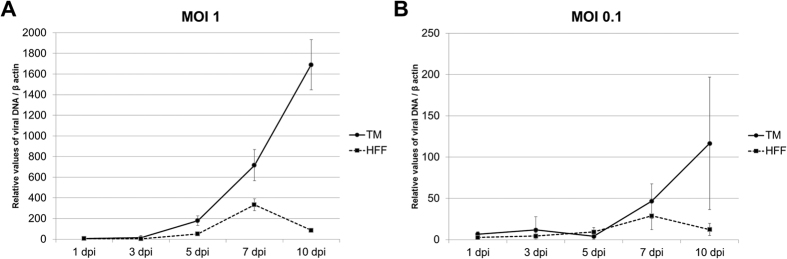
Viral DNA accumulation after human cytomegalovirus infection in human trabecular meshwork cell (TM) and human foreskin fibroblast cells (HFF). Cells were infected with CMV AD169 at a high multiplicity of infection (MOI; 1) (**A**) and at a low MOI (0.1) (**B**). Cells were harvested at 1, 3, 5, 7 and 10 day after infection (dpi) and viral DNA was extracted from cells and processed for qPCR analysis of viral DNA accumulation (UL26). Real-time PCR with β-actin primers was performed to serve as an internal control for input DNA. Data are the averages of three independent DNA samples from the infected cells. Values are mean ± standard error.

**Figure 4:**
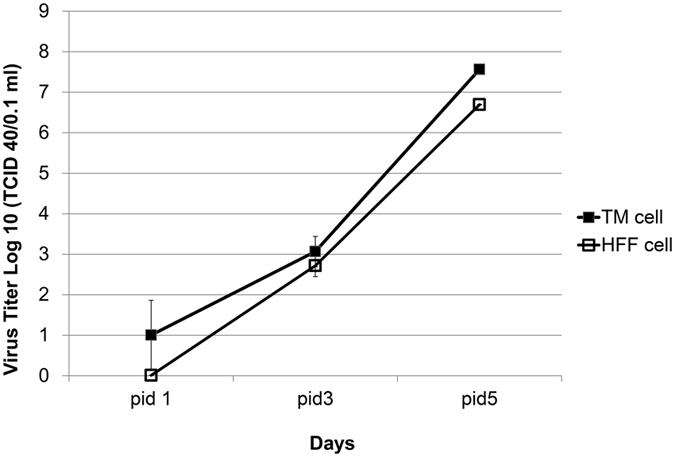
Cytomegalovirus (CMV) replication in human foreskin fibroblast (HFF) and human trabecular meshwork cells. Cells were incubated with CMV at an multiplicity of infection of 1. After a 2 hour incubation period, inoculum was removed, and cells were washed 3 times and fed maintenance media. At varying times between 1 day post-infection (dpi) 1 and 5, supernatant was removed and assayed for infectious virus on HFF cells. Results are expressed as the mean + /− standard error of the mean for experiments performed in triplicate.

To determine whether human TM cells support CMV infection, human TM cells and human foreskin fibroblast (HFF) cells were cultured in the presence of high or low MOI (and Mock infection, data not shown) and examined at 1, 3, and 5 dpi. A subset of samples were fixed and immunostained (F-actin, IE antigen, and DAPI) to demonstrate the productive infection of cells. After CMV infection in TM cells, the IE antigen was detected at 1 dpi, and it was well observed at 3 and 5 dpi at high (Fig. 1) and low MOI (data not shown). Consistent with this, in CMV infection in HFF cells, the IE antigen was detected at 1 dpi and was increased at 3 and 5 dpi at high (Fig. 2) and low MOI (data not shown). Next, we performed quantitative real-time PCR to determine viral DNA replication. A marked increase of viral DNA was noted in the first 10 days in TM cells; however, in HFF cells, the production of viral DNA was increased until 7 dpi and subsequently decreased at 10 dpi (Fig. 3). Growth curves of virus over time show CMV replication in TM cell in comparison with HFF cells (Fig. 4). The virus production in TM cells corresponded closely to the HFF cells in 1 and 3 dpi at high MOI. However, an approximately 10-fold higher level of viral replication in TM cells compared with that in HFF were noted in 5 dpi (*P* < 0.001).

### Virus-induced cytopathology in human TM cells

**Figure 5:**
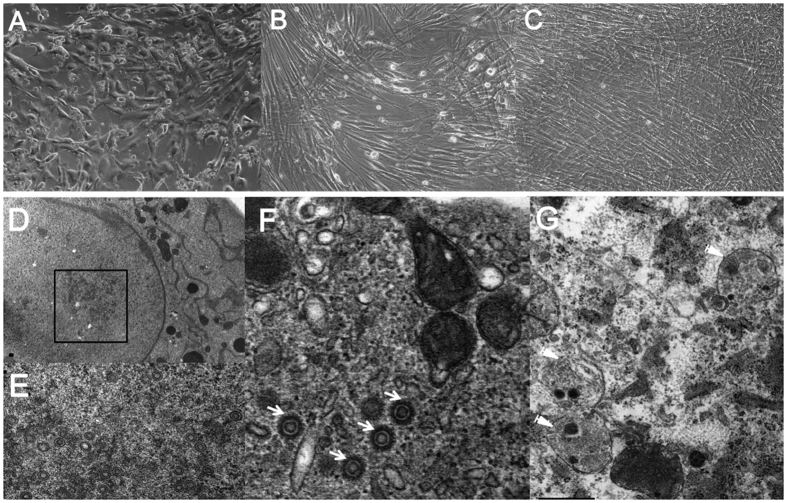
Human cytomegalovirus-induced cytopathology in human trabecular meshwork cells on phase-contrast microscopy (**A–C**) and on electron microscopy (**D–G**) at 5 days after CMV infection. TM cell at MOI of 1 (**A**), at MOI of 0.1 (**B**), and normal uninfected TM cells (**C**) are seen (magnification 100x). Electron microscopy image shows large numbers of viral capsids in the nucleus of the TM cells (**D,E**). Also, homogeneous masses of electron-dense material and viral particles comprise the cytoplasmic iniclusion. Magnification of viral capsids observed in the nucleus (**F**, white arrow) double-membraned autophagosomes (white triangle) are seen (**G**). Bar = 200 nm.

The typical CMV cytopathic effect of ballooned and swollen cells was noted in 100% of human TM cells by 5 dpi (Fig. 5A–C). Immunocytochemical staining revealed that compared with Mock infection, CMV infection induced disorganization, decreased the number of stress fibers, and increased the swollen appearance of cells (Fig. 1B).

Electron microscopy of human TM cells after CMV infection showed a large number of viral capsids in the nucleus at 5 dpi (Fig. 5D–F). In the cytoplasm, homogeneous masses of electron-dense material and viral particles were noted. Double-membraned autophagosomes along with viral capsids were also noted (Fig. 5G).

### CMV-induced elevation of TGF-β1 in TM cells

**Figure 6:**
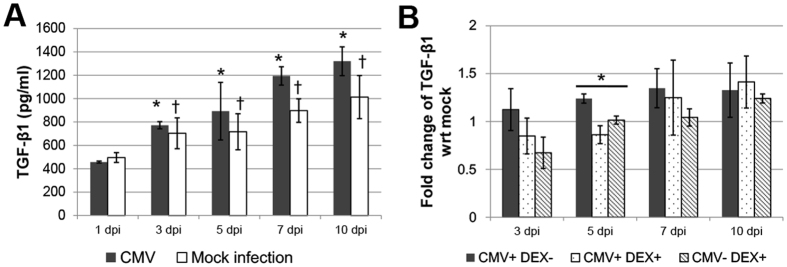
CMV infection in human TM cells induces active TGF-β1 production. After CMV infection at a high MOI, supernatants were assayed for total TGF-β1 production using a TGF-β1 responsive luciferase bioassay. (**A**) Expression of TGF-β1 was increased 1 dpi (*P < 0.05 vs. TGF-β expression 1 dpi; ^†^*P* < 0.05 vs. TGF-β expression of the Mock infection). (**B**) Treatment with DEX significantly decreased the production of TGF-β1 5 dpi (*P < 0.05 for TGF-β1 expression of CMV infection without DEX, CMV infection with DEX, and DEX treatment alone). Results are expressed as the mean ± standard deviation of three different experiments. DEX, dexamethasone.

To ascertain the effects of CMV infection on outflow facility, the expression of TGF-β1 in human TM cells was measured. The expression of TGF-β1 at 3, 5, 7, and 10 dpi was significantly increased compared with that at 1 dpi (*P* < 0.05). Compared with Mock infection, CMV infection significantly increased the expression of TGF-β1 at each time point (all *P* < 0.05, Fig. 6A). Next, to investigate the mechanism of action of corticosteroids on viral anterior uveitis, TM cells were examined at baseline and after CMV infection, in the presence or absence of DEX. Thus, effects of CMV alone, DEX alone, or CMV and DEX together were compared to TM cells at baseline. Treatment with DEX decreased the production of TGF-β1, and this effect was significant at 5 dpi (Fig. 6B).

### Expression of FN, COL1A, α-SMA, and CTGF induced by CMV and DEX

**Figure 7:**
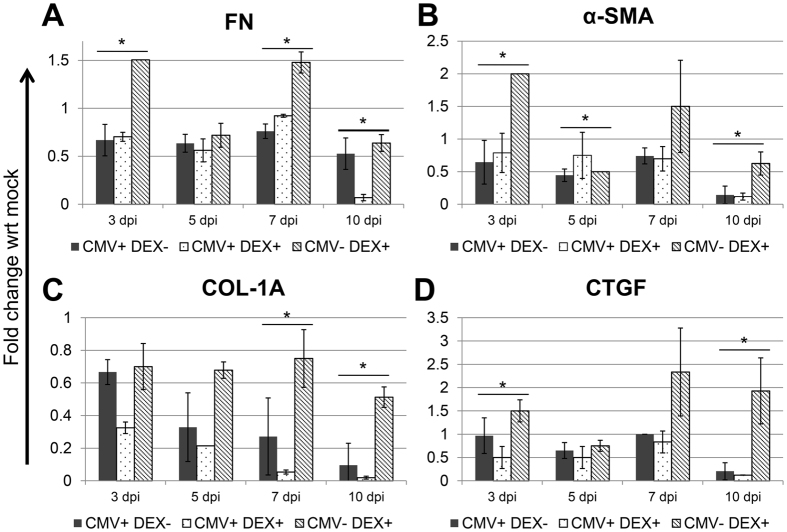
Quantitative determination of mRNA expression levels according to CMV infection and DEX treatment. (**A**) Expression of fibronectin (FN), (**B**) α-smooth muscle actin (α-SMA), (**C**) collagen (COL)-1A, and (**D**) connective tissue growth factor (CTGF). Transcripts from cells under various experimental conditions were compared to those produced at baseline by uninfected and unstimulated TM cells.

To characterize the effect of CMV on the elevation of IOP in viral anterior uveitis, RNA was extracted from cell lysates, and real-time PCR was performed using a commercial PCR array to detect transcripts associated with the elevation of IOP (ECM molecules (fibronectin (FN), collagen (COL)1A), α-smooth muscle actin (SMA), and connective tissue growth factor (CTGF)). Transcripts from cells under various experimental conditions were compared to those produced at baseline by uninfected and unstimulated TM cells. Compared with uninfected cells, CMV-infected cells showed decreased induction of many mRNA transcripts encoding fibrogenic matrix proteins (FN, COL1A, and α-SMA) (Fig. 7A–C). In contrast, after exposure to DEX, transcriptional up-regulation of FN-1, α-SMA, and CTGF was observed (Fig. 7A,C,D), whereas the transcription of COL1A was reduced (Fig. 7C). After CMV infection in the presence of DEX, the transcription of these fibrogenic molecules was reduced compared with those exposed to DEX alone.

### Effect of Ganciclovir in CMV infection

**Figure 8:**
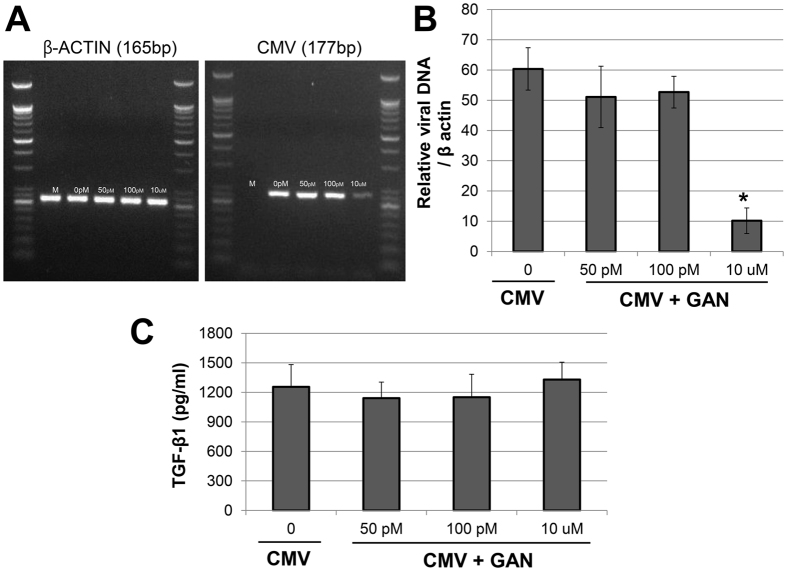
Effect of ganciclovir on viral DNA accumulation (**A**,**B**) and induction of TGF-β1 production after human cytomegalovirus infection in human trabecular meshwork cells (**C**). Cells were harvested at 5 day post-infection at a high multiplicity of infection (MOI 1) under the treatment with different concentrations of ganciclovir (GAN). Treatment with 10 μmol of ganciclovir significantly decreased the viral DNA accumulation (p < 0.001) (**A,B**). However, treatment with ganciclovir did not affect the TGF-β production using a TGF-β1 luciferase bioassay. Results are expressed as the mean +/− standard deviation of three different experiments.

When TM cells were exposed to different concentration of ganciclovir, we found that 10 μmol of ganciclovir significantly decreased the viral DNA accumulation (Fig. 8A,B, *P* < 0.001). However the treatment with ganciclovir did not significantly affect the TGF-β1 production compared with those exposed CMV alone (Fig. 8C).

## Discussion

Herpes viruses are common infectious causes of hypertensive anterior uveitis^2^,^7^,^15^. Herpes virus infection in the anterior chamber has been detected in patients diagnosed with Posner–Schlossman syndrome and Fuchs heterochromic iridocyclitis, which were previously believed to be idiopathic^5^,^18^. Particularly, CMV is associated with corneal decompensation and a greater requirement for glaucoma surgery. Regarding corneal complications associated with CMV, Hosogai *et al*.^19^ reported that CMV can replicate in a human corneal endothelial cell culture model. Considering that herpes virus induces high IOP elevation coinciding with the duration of uveitis, it is assumed that the herpes virus infects TM cells, which are the key cells involved in IOP regulation. However, to our knowledge, the permissive replication of CMV in human TM cells or pathophysiological changes of TM induced by CMV infection have yet to be reported.

### Replication of CMV in human TM cells

An owl’s eye appearance, which is characteristic of CMV-infected cells, has been observed in patients with CMV corneal endotheliitis^20^. In this study, the expression of IE viral gene products, a gene expressed 0–4 h after infection and involved in the regulation of transcription^21^, occurred in human TM and HFF cells (Figs 1 and 2) from 1 dpi. Indeed, the production of viral DNA increased significantly, up to 10 dpi in human TM cells (Fig. 3), whereas the amount of viral DNA showed no increase after 7 dpi in HFF cells. Also, replication of infectious viruse in TM cells corresponded closely to the HFF cells (Fig. 4).

CMV infections are characterized by a marked restriction of susceptible cells^22^. *In vitro* studies usually rely on the productive replication of CMV in HFF cells. The efficiency of CMV replication depends on the maturity level of the cells^23^,^24^. To be permissive for CMV, cells need to reach a certain stage of differentiation for the sequential expression of viral genes (IE, E, and L genes)^10^. In this regard, the productive infection of CMV in human TM cells suggests that TM cells are at a stage of differentiation to be permissive. Consistent with this hypothesis, TM cells possess similarities with human mesenchymal stem cells, expressing notch1, sox, and similar cell surface molecules, and suppressing T-cell proliferation^25^.

**Figure 9:**
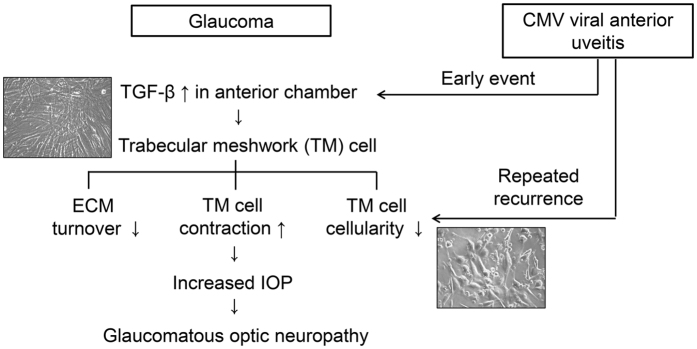
Putative events leading to glaucomatous optic neuropathy following CMV viral anterior uveitis. Elevated TGF-β in anterior chamber of glaucoma patient is considered to be main causative and a result of glaucomatous pathology, and its main target is the trabecular meshwork cell. TGF-β1 and TGF-β2 increase extracellular matrix (ECM) production and inhibit the degradation of presenting ECM^8^. TGF-β1 increases the contraction of TM cell and TGF-β2 decreases the population of TM cell by inhibition of cell proliferation. These changes contributes to outflow resistance, leading to elevation of intraocular pressure (IOP), which eventually, leads to glaucomatous optic neuropathy. In CMV viral anterior uveitis, virus produces high amount of TGF-β1 as an early event, which may increase the concentration of TGF-β1 in the anterior chamber. With the repeated recurrence, the decreased cellularity of TM cells induced by CMV infection may cause the elevation of IOP and poor prognosis of the patients.

In this study, CMV infection in human TM cells produced cytopathic changes, including swelling of the cells, disorganization, and a decrease in the number of stress fibers (Figs 1 and 5A–C). Contraction of stress fibers in human TM cells leads to higher rigidity and an increase in outflow resistance (Fig. 9)^8^. Therefore, the disorganization and reduced stress fibers induced by CMV infection may counteract IOP elevation. In addition, multiple double-membraned autophagosomes were noted in electron microscopy analyses (Fig. 5G). The infection of HFF with human CMV or HSV increased autophagy in the early stage of infection^26^. Autophagy is a process by which cellular organelles and abnormal or foreign proteins are degraded to maintain cell viability in times of stress. It has been shown that senescence of human TM cells is associated with oxidative stress and an increase in autophagy^27^. Further studies are required to investigate the relationship between autophagy and TM cell changes induced by CMV infection.

### TGF-β1 is elevated in human TM cells in a CMV infection model

In this study, we demonstrated that CMV infection resulted in significantly elevated expression of TGF-β1 in human TM cells during the first 10 days after infection (Fig. 6A). TGF-β1 is an upstream cytokine that regulates outflow facility by inducing characteristic structural changes of TM cells. CMV infection is known to induce its secretion in infected fibroblasts, astrocytes, and osteosarcoma cells^10^,^11^,^28^. TGF-β1 is expressed at higher levels in HCMV-infected renal allografts compared with uninfected allografts, and these higher TGF-β1 levels are associated with renal allograft rejection^12^. In hypertensive anterior uveitis, CMV infection is known to be associated with a greater requirement for glaucoma surgery^7^. Our study suggests that the acute elevation of TGF-β1, as well as the decreased cellularity of TM cells induced by CMV infection, caused the elevation of IOP and poor prognosis of the patients.

Interestingly, treatment with DEX decreased TGF-β1 levels for the first 5 days after infection, although this effect was reduced at 7 and 10 dpi (Fig. 6B). This seems to explain the prompt restoration of viral anterior uveitis with conventional corticosteroids and antiglaucoma agents in clinical settings. Flugel-Koch *et al*.^29^ reported that DEX increased the expression of thrombospondin-1, which is involved in the activation of TGF-β in human TM cells. In this regard, treatment with DEX decreased TGF-β1 during the early stage of infection, but eventually, TGF-β1 levels increased with a longer duration of DEX treatment.

Although corticosteroids are the current treatment for viral anterior uveitis, steroid-induced iatrogenic glaucoma is a frequent and potentially debilitating side effect. The underlying pathophysiology of steroid-induced glaucoma is understood to include increased resistance to outflow with increased deposition of ECM proteins in TM tissues^30^. Consistent with this, we found that mRNA transcripts encoding fibrogenic matrix proteins (FN, α-SMA and CTGF) had increased after exposure to DEX, whereas cells infected with CMV produced a lower amount of mRNA transcripts encoding the same fibrogenic matrix proteins (Fig. 7A,B,D). Despite the prompt IOP-lowering effect of corticosteroid treatment in hypertensive anterior uveitis, its long-term use may eventually elevate IOP by increasing resistance in aqueous outflow, as shown in this study. Furthermore, the use of steroids in infectious diseases remains controversial, as steroids are immunosuppressive agents that may potentially increase virus recurrence rates with chronic use. Steroids inhibit the NF-κB signaling pathway that regulates innate immunity, an integral host process that limits viral pathogenesis^31^. Further studies are warranted to further elucidate the role of steroids in the productive infection of CMV.

Finally, treatment with viral DNA polymerase inhibitor ganciclovir did not affect the TGF-β1 production, although it decreased viral DNA replication (Fig. 8). In the study of Shimamura *et al*.^32^, antiviral agent did not prevent TGF-β1 activation after epithelial to mesenchymal transition after human CMV infection in renal tubular epithelial cells. They speculated that TGF-β1 production may occur as an early event, preceded viral replication as represented by viral polymerase inhibition. In this regard, TGF-β1 production in the early phase of CMV infection seems to be associated with the acutely elevated IOP with the concurrent anterior chamber inflammation. Clinically, the use of antiviral agent is known to lower the recurrence rate of the viral anterior uveitis. However, the use of antiviral agent alone cannot lower the IOP nor change the course of the disease. It may be associated with the fact that TGF-β production cannot be inhibited by antiviral agent. Therefore, developing treatments targeting the TGF-β pathway in clinical settings may be therapeutically beneficial for the management of viral anterior uveitis.

### Summary

The data presented in this study definitively demonstrate that human TM cells effectively support CMV replication. This suggests that active viral infection in human TM cells could be the key mechanism underlying the elevation of IOP in anterior viral uveitis (Fig. 9). Importantly, we demonstrated that CMV infection enhanced TGF-β1 production in human TM cells and that this increase was countervailed by treatment with corticosteroids. This *in vitro* study provides a potential pathogenic mechanism for the observed associations between CMV infection and elevation of IOP in viral anterior uveitis.

## Materials and Methods

### Cells

Primary HFF monolayers were propagated and maintained as described previously^33^. Primary human TM cell cultures were derived from two separate normal cadaver eyes (aged 39 and 16 years). All eyes were enucleated within 6-h postmortem and tissues were explanted for culture within 24-h postmortem. TM cells were grown in tissue culture as described previously^34^. Maintenance growth medium consisted of low glucose Dulbecco’s modified Eagle’s medium (DMEM) supplemented with 15% fetal bovine serum (FBS; Invitrogen-Gibco, Grand Island, NY, USA), fibroblast growth factor (FGF)-2 (1 ng/mL), and 1% penicillin-streptomycin. For some experiments, primary TM cells obtained from ScienCell Research Labs (Carlsbad, CA, USA) were used and cultured to 100% confluence in Trabecular Meshwork Cell Medium (catalog No. 6591; ScienCell Research Labs)^35^. TM cells from the fourth or fifth passage were used. For each experiment, TM cultures were seeded into 12-well plates and allowed to grow to confluence at 37 °C in a 5% CO_2_ atmosphere.

### Viruses, Dexamethasone and Ganciclovir treatment

HFF monolayers were used to propagate CMV strain AD169 (passage number 2). Infected cultures were harvested by freezing and thawing one time, followed by centrifugation for 20 min at 2,000 rpm. Supernatant fluids were used as virus inoculum. Cell-free CMV was collected by filtration of the infected cell medium or extract through a 0.22-μm filter. After reaching confluence, TM and HFF cells were incubated with the virus stock preparation for a 2-h adsorption period at 37 °C in 5% CO_2_ with a multiplicity of infection (MOI) of 1 and 0.1, respectively. After removal of the viral inoculum, the infected cells were washed once with 1 × phosphate-buffered saline (PBS) and growth medium was applied^36^. To ensure viability of the virus stock, HFFs were infected in parallel. Phase contrast microscopy confirmed that confluent cultures formed a monolayer with the typical morphological cytopathic changes related to CMV infection. In some experiments, TM cell cultures were exposed to 100 nM dexamethasone (DEX, Sigma-Aldrich Corp., St. Louis, MO, USA) and equivolume treatments of ethanol (EtOH) as the vehicle on the following day of infection^37^. To assess the effect of ganciclovir on the viral DNA accumulation and TGF-β1 production, TM cell cultures were exposed to 50 pmol, 100 pmol, and 10 μmol ganciclovir (Selleckchem, Houston, TX, USA).

### Immunohistochemistry

To observe cytopathic effects, infected and uninfected TM and HFF cells were fixed in 4% buffered formalin. The immediate-early (IE) antigens were analyzed by immunofluorescence imaging. Formaldehyde-fixed cells were treated with 0.1% trypsin for 20 min at 37 °C, and non-specific binding was blocked with 2.5% normal horse serum (RTU Vectastain Universal Elite ABC Kit, Vector Laboratories, Burlingame, CA, USA) for 30 min. A pooled murine monoclonal anti-CMV IE antibody (Anti-CMV Immediate Early Antigen Antibody LS-C103255, LSBio, Seattle, WA, USA) was applied (1:50 dilution) in 2.5% normal horse serum overnight at 4 °C, and a secondary goat anti-mouse antibody (VectaFluor R.T.U. DyLight 488 Anti-Mouse, Burlingame, CA, USA) was subsequently applied for 1 h. Rhodamine phalloidin (Invitrogen, Carlsbad, CA, USA) was used to visualize stress fiber structures. Cells mounted in Vecta-Stain mounting media (VECTASHIELD^®^ with DAPI, Burlingame, CA, USA) were observed with inverted fluorescence microscopy (IX83, Olympus, Corporation, Tokyo, Japan).

### Viral DNA replication assays

Viral DNA was harvested at 1, 3, 5, 7, and 10 days post-infection (dpi) and isolated from the cells in 12-well plates in triplicate using the Qiagen column (QIAmp^®^ DNA Mini Kit; Qiagen, Hilden, Germany). The replicated viral DNA was quantitated by real-time polymerase chain reaction (PCR) using HCMV UL26 primers and probes as described previously^38^. Real-time PCR with β-actin primers was also performed to serve as an internal control for input DNA. Data are averages of three independent experiments. The sequences of the primer sets are shown in Table 1.

### Assay for Infectious Virus

Medium (0.1 ml) from CMV –exposed TM cultures was inoculated into monolayers of HFF cells and incubated for 2 hours at 37 °C. Virus titers were assessed using 50% tissue culture infectious dose (TCID_50_) assay with serially diluted supernatants in quadruplicate infection in 48-well culture dishes using the method of Reed and Muench.

### Electron microscopy

The infected cells were fixed with a fixative containing 2.5% glutaraldehyde in 0.1 M sodium cacodylate buffer (pH 7.4). Ultrathin sections (0.1-μm-thick) were cut and mounted on grids coated with Formvar and were examined with a transmission electron microscope (model 1200EX; JEOL, Tokyo, Japan).

### ELISA for TGF-β

Levels of secreted TGFβ-1 were measured by determining its concentration in conditioned medium using a commercially available sandwich enzyme-linked immunosorbent assay (ELISA, Total TGF-β1 ELISA Kit with Pre-coated Plates; BioLegend, San Diego, CA, USA). Conditioned medium was harvested on days 1, 3, 5, 7, and 10, cleared by centrifugation, and stored at −70 °C. Conditioned medium was acid-activated and directly assayed using an ELISA plate reader at 450 nm, according to the manufacturer’s instructions. Protein concentrations were calculated from a standard curve with two-fold serial dilutions and a highest standard of 500 pg/mL.

### RNA extraction and real-time PCR

Total cellular RNA was extracted from cultured TM cells (RNeasy Mini Kit; Qiagen, Valencia, CA), and a cDNA synthesis kit (PrimeScript™ RT reagent Kit, Takara, Japan) was used for first-strand cDNA synthesis. The primer pairs are listed in Table 1. The relative expression levels of mRNA were determined using a Roche Diagnostics LightCycler^®^ 2.0 Real-Time PCR System (Roche GmbH, Mannheim, Germany). A total volume of 20 μL of reaction mix was subjected to the following PCR program: 1 step at 95 °C for 10 min for initial denaturation followed by 1 step of 40 cycles each at 95 °C for 1 0 s, 60 °C for 10 s, and 72 °C for 15 s, and a denaturation cycle for creation of a melting curve. Each reaction mixture consisted of 2 μL of reaction master mix (LightCycler^®^ FastStart DNA Master SYBR Green I; Roche, Mannheim, Germany), 3 mM MgCl_2_, 1 μL of each primer (10 μM) and 2 μL of template cDNA. Reactions for each sample were run in triplicate, cycle thresholds (Ct) were normalized to β-actin expression, and comparative quantitation was performed (LightCycler^®^ software 4.1, Mannheim, Germany). Only individual PCR samples with single-peak dissociation curves were selected for data analysis.

### Statistical analysis

Experiments were performed in triplicate and representative results are shown. The Student’s t-test was performed for comparisons between two groups. One-way analysis of variance (ANOVA) was used for comparison of results between three groups. A value of *P* < 0.05 was considered to indicate statistical significance.

## Additional Information

**How to cite this article:** Choi, J. A. *et al*. Enhanced cytomegalovirus infection in human trabecular meshwork cells and its implication in glaucoma pathogenesis. *Sci. Rep.* **7**, 43349; doi: 10.1038/srep43349 (2017).

**Publisher's note:** Springer Nature remains neutral with regard to jurisdictional claims in published maps and institutional affiliations.

## Figures and Tables

**Table 1:** Sequence for forward and reverse primer sets used for real-time RT-PCR and real-time PCR.

Amplification	Forward primer	Reverse primer
UL26	5′-AACATCGCGTCGGTGATTTCTTGC-3′	5′-ACAGCTACTTTGAAGACGTGGAGC-3′
Fibronectin	5′-CTGGCCGAAAATACATTGTAA-3′	5′-CCACAGTCGGGTCAGGAG-3′
α-SMA	5′-GACAATGGCTCTGGGCTCTGTAA-3′	5′-CTGTGCTTCGTCACCCACGTA-3′
Collagen1A	5′-GGAATGAAGGGACACAGAGG-3′	5′-TAGCACCATCATTTCCACGA-3′
CTGF	5′-CTCCTGCAGGCTAGAGAAGC-3′	5′-GATGCACTTTTTGCCCTTCTT-3′
β-actin	5′-GTCCACCTTCCAGCAGATGT-3′	5′-AAAGCCATGCCAATCTCATC-3′

α-SMA: smooth muscle actin; CTGF: connective tissue growth factor.
